# Clinical and Oncological Value of Preoperative BMI in Gastric Cancer Patients: A Single Center Experience

**DOI:** 10.1155/2015/810134

**Published:** 2015-02-10

**Authors:** Costantino Voglino, Giulio Di Mare, Francesco Ferrara, Lorenzo De Franco, Franco Roviello, Daniele Marrelli

**Affiliations:** ^1^Department of Medicine, Surgery and Neurosciences, Unit of General Surgery and Surgical Oncology, University of Siena, Policlinico “Le Scotte”, Viale Bracci, 53100 Siena, Italy; ^2^Department of Medicine, Surgery and Neurosciences, Unit of General and Mini-Invasive Surgery, University of Siena, Policlinico “Le Scotte”, Viale Bracci, 53100 Siena, Italy

## Abstract

*Introduction*. The impact of preoperative BMI on surgical outcomes and long-term survival of gastric cancer patients was investigated in various reports with contrasting results. *Materials & Methods*. A total of 378 patients who underwent a surgical resection for primary gastric cancer between 1994 and 2011 were retrospectively studied. Patients were stratified according to BMI into a normal group (<25, group A), an overweight group (25–30, group B), and an obesity group (≥30, group C). These 3 groups were compared according to clinical-pathological characteristics, surgical treatment, and long-term survival. *Results*. No significant correlations between BMI and TNM (2010), UICC stage (2010), Lauren's histological type, surgical results, lymph node dissection, and postoperative morbidity and mortality were observed. Factors related to higher BMI were male gender (*P* < 0.05), diabetes (*P* < 0.001), and serum blood proteins (*P* < 0.01). A trend to fewer lymph nodes retrieved during gastrectomy with lymphadenectomy in overweight patients (B and C groups) was observed, although not statistically significant. There was no difference in overall survival or disease-specific survival between the three groups. *Conclusion*. According to our data, BMI should not be considered a significant predictor of postoperative complications or long-term result in gastric cancer patients.

## 1. Introduction

Gastric cancer represents one of the most frequent neoplasia worldwide, and specifically the fourth and fifth most common cancer in men and women and the third and fifth cause of cancer-related death [1]. To date the evidence shows a rising incidence of obesity, in particular in Western Countries, and an increased relative risk of cancer-related mortality at multiple sites with increased BMI [2]. Some authors suggest a relationship between increased BMI and esophageal and gastric cardia adenocarcinoma [3–5]. Some theories try to explicate the etiology of this relationship, as it seems to be related to the metabolic syndrome and the relative chronic inflammation [6] or to the higher abdominal pressure and the resulting gastroesophageal reflux [7, 8], but actually no clear etiology has been established between obesity and gastric carcinogenesis. Usually obese cancer patients have often been perceived as being at high risk of surgical complications. In fact, there are several technical disadvantages during a surgical procedure for obese patients, including poorer surgical visibility, blood oozing from soft tissues, a dissection plane hindered by adipose tissue, and difficulty with anastomosis. Another important aspect in the intricate relation between obesity and gastric cancer is the effect of BMI on the patient's outcomes following surgical gastric resection for adenocarcinoma. Many authors investigated this relation, with very different results in terms of survival, pathological findings, and results of surgical procedures [9–11]. The aim of this study was to examine the relationship between overweight and long-term survival of patients undergoing gastrectomy for gastric cancer in our center and to evaluate the postoperative complications and the adequacy of surgical therapy.

## 2. Materials and Methods

In this study 378 patients with primary gastric cancer surgically treated in the Unit of General Surgery and Surgical Oncology, University of Siena, between 1994 and 2011 were included. The histological confirmation of the neoplasia was preoperatively achieved by endoscopic biopsies. In order to perform the most appropriate surgical treatment an accurate preoperative staging was performed in all patients by computed tomography (CT) scan and, when necessary, by endoscopic ultrasound. Subtotal or total gastrectomy was performed according to tumor location and the possibility to obtain negative resection margins and a potentially curative (R0) resection. The extent of lymphadenectomy was classified according to the Japanese Gastric Cancer Association (JGCA) guidelines, as previously described [12, 13]. All the specimens were analyzed for pTNM determination. Tumor stage was defined according to the pathological tumor node metastasis (pTMN system) classification proposed by the International Union against Cancer (UICC/AJCC, 7th edition, 2010). All cases before 2010 were revised and the pTNM classification has been updated at the 7th edition [14]. Gastroesophageal junction tumors were defined according by the classification described by Siewert et al. [15], and type I tumors (distal esophageal) were excluded. Patients were stratified on the basis of BMI into three groups: normal group (BMI < 25 kg/m^2^, group A), overweight group (BMI 25–30 kg/m^2^, group B), and obesity group (BMI ≥ 30 kg/m^2^, group C). Clinical characteristics, surgical procedures, and histological findings were recorded in a specific database. Data were compared between the three groups, with special reference to postoperative outcome, morbidity, mortality, and long-term survival. Statistical analysis was performed with the *χ* ^2^ test or Fisher exact test to compare categorical variables. The Mann-Whitney *U* test and Kruskal-Wallis one-way analysis of variance (ANOVA) were used to compare continuous variables not normally distributed. Cumulative survival was calculated by the life table method of Kaplan and Meier, and the log-rank test was used to distinguish significant differences. Statistical significance was determined at *P* value of <0.05.

## 3. Results

218 patients were selected in group A, 121 in group B, and 39 in group C. The median BMI was 24.03 kg/m^2^ and the median age was 66 years. We performed a subtotal gastrectomy in 257 cases and a total gastrectomy in 121. In total, a median number of 35 lymph nodes (range: 3–140) was removed. Information regarding BMI, gender, age, and surgical data was available for all patients, whereas data regarding pT, pN, and tumor site were missing for some cases.

Clinicopathological features of the patients are summarized in Table 1.

**Figure 1:**
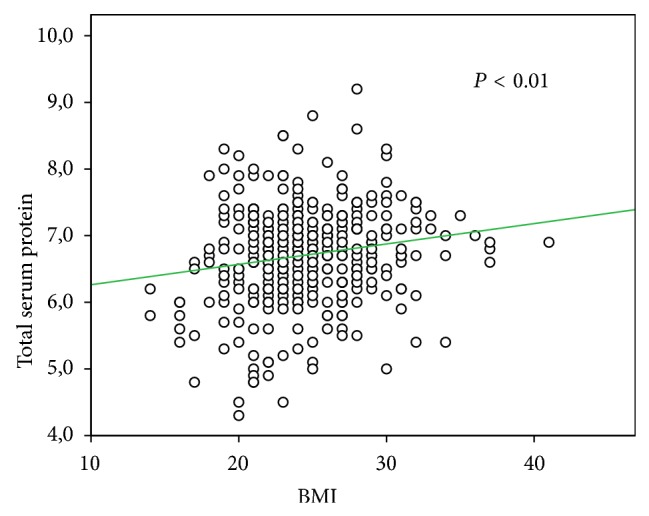
Correlation between BMI and serum blood protein (*P* < 0.01).

**Figure 2:**
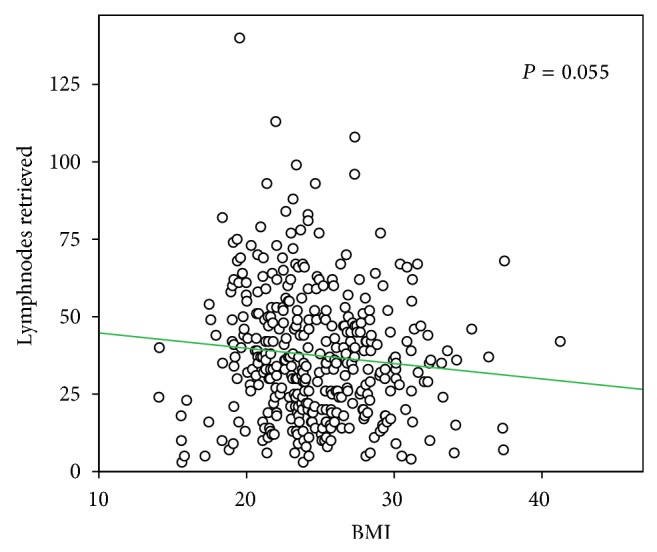
Correlation between BMI and number of lymph nodes harvested (*P* = 0.055).

Preoperatively, higher BMI was associated with male gender (*P* < 0.05), type 2 diabetes (*P* < 0.001) (Table 1), and serum blood proteins (*P* < 0.01) (Figure 1). No significant relationships were found between BMI and TNM (2010), UICC stage (2010), Lauren's histological type, tumor site, major morbidity, and hospital stay. A trend to fewer retrieved lymph nodes was observed in overweight patients (B and C groups), as the median number of lymph nodes examined was 37 for group A (range 3–140), 33 for group B (range 5–108), and 35 for group C (range 4–68) (*P* = 0.059 Kruskal-Wallis). This difference was also not statistically significant when correlation analysis was used (*P* = 0.055) (Figure 2). The median number of metastatic lymph nodes was 4 for group A (range 0–86), 3 for group B (range 0–55), and 5 for group C (range 0–63) (*P* = 0.586).

**Figure 3:**
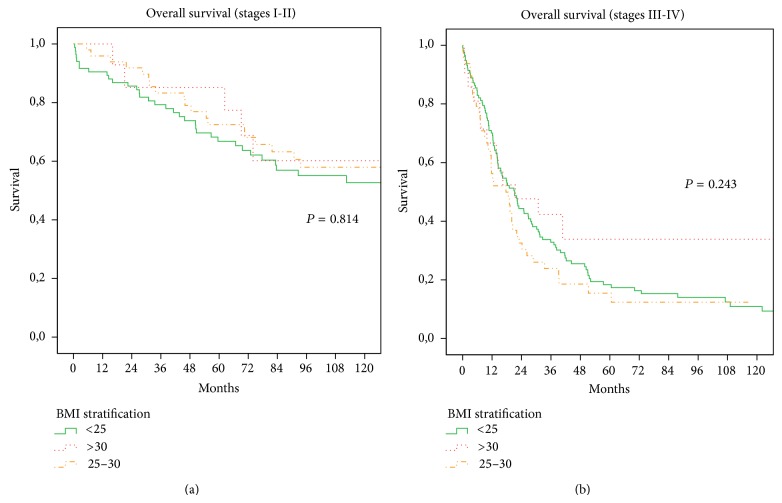
(a)-(b) Overall survival for overweight group, obesity group, and normal group according to tumour stage. (a) Stages I and II (*P* = 0.814 log-rank test). (b) Stages III and IV (*P* = 0.243 log-rank test).

**Figure 4:**
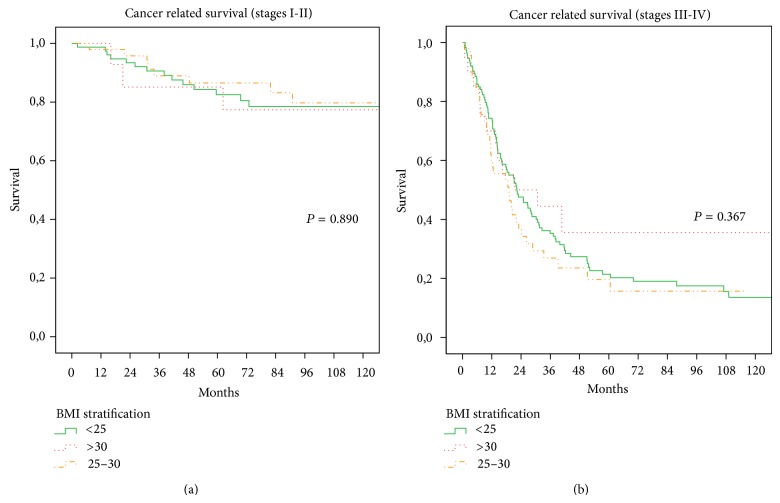
(a)-(b) Cancer related survival for overweight group, obesity group, and normal group according to tumour stage. (a) Stages I and II (*P* = 0.890 log-rank test). (b) Stages III and IV (*P* = 0.367 log-rank test).

The rates of postoperative complications in the A, B, and C groups were 28.9%, 31.4%, and 33.3%, respectively. These differences did not reach statistical significance (*P* = 0.803). The rate of patients who died during postoperative hospital stay was 5.0% in group A, 1.7% in group B, and 2.6% in group C, and the difference was not significant (*P* = 0.230). In this analysis, median follow-up was 35 months (range 1–199). No statistical differences were found between the obesity, overweight, and normal groups. The 10-year overall survival rates were 28% in group A versus 36% in group B versus 44% in group C (*P* = 0.457). The 10-year disease specific survival rates were 40%, 50%, and 55% in group A, B, and C patients, respectively (*P* = 0.42). The overall survival and disease specific survival, after stratification based on tumor staging (I-II, III-IV), are shown in Figures 3 and 4; no significant differences were also observed in this stratified analysis according to BMI groups.

## 4. Discussion

The BMI is a measure for human body shape based on an individual's weight and height. It is well known that obesity is associated with cancer incidence and mortality; however the role of high BMI in cancer survival is less well understood.

The prevalence of overweight and obesity in the United States rose from the 1960s to 2010 [16]. The obesity and overweight rate was 45.8 per cent in Italy, 63.1 per cent in United States, and only 23.3 per cent in Korea. In fact, the mean or median BMI in Asian population is lower than that observed for white or European populations, but they have a higher percentage of body fat than white people of the same age, sex, and BMI. Furthermore, white people in Europe generally have a higher percentage of body fat for the same BMI than do those in USA [17]. Obesity is associated with a poor overall and disease free survival or advanced stage in multiple malignancies, as gastric [3], pancreatic [18], breast [19], and colorectal cancer [20]. Several recent studies have investigated the relationship between BMI and surgical outcomes of gastric cancer patients, with contrasting results in terms of operating time, number of dissected lymph nodes, postoperative morbidity and mortality, hospital stay, and long-term survival [21–24] (Table 2). This rather low originality may be considered a limitation to the present paper. However, many of these studies come from Eastern Countries, and few experiences from Western centers specialized in extended lymphadenectomy have been reported in literature.

The technical difficulties due to obesity in many abdominal surgical procedures have been described, and obesity has been associated with a higher risk of perioperative morbidity after gastric cancer surgery in some series [25–27]. Obesity is related to a greater incidence of comorbidities, such as hypertension and diabetes mellitus, and this could justify, in part, these results. In our experience, we found no significant association between BMI and perioperative complications, morbidity, and mortality. Other authors, in Western and Eastern series, obtained the same results [9, 28–30]. In our opinion, this may be due to the high experience of our center in extended lymphadenectomy, which has been introduced as a standard treatment for gastric cancer several years ago [12, 13]. As other authors [29, 31] we did not find any relationship between increased BMI and any of the cancer-related parameters such as tumor site, Lauren classification, TNM stage, and metastatic lymph nodes. Additionally, we found no significant difference in the number of retrieved lymph nodes between normal, overweight, and obesity groups. According to the National Comprehensive Care Network (NCCN) guidelines [32], which recommend examination of 15 lymph nodes or more for adequate staging, we retrieved a median number of 35 lymph nodes. Even if BMI was not statistically related with the number of harvested lymph nodes, a trend to fewer retrieved lymph nodes in obese and in overweight patients was observed. This could indicate the isolation of lymph nodes from the specimen with abundant fat might be more difficult than their retrieval in nonobese patients. In the literature very discordant data are reported, in particular in terms of race and geographic origin; European [9, 27] and American authors [29, 33] described an inverse relationship between harvested lymph nodes and BMI, whereas Asiatic authors showed contradictory results [10, 26, 30, 31]. It is relevant, in our opinion, that despite the high number of removed lymph nodes, no significant differences in postoperative morbidity and mortality according to preoperative BMI were observed in the present study.

As a confirmation of the oncological effectiveness of lymphadenectomy in overweight patients, in our study we did not find any statistical difference between groups in terms of overall and disease free survival, in early as well as advanced stage of disease. In the literature different results are reported [9, 10, 23, 25, 26, 29–31, 34–36]. However, the general opinion is that increased BMI does not affect long-term outcomes of gastric cancer patients. In our experience long-term outcome of patients with high BMI was similar to or even better than other patients. This may underline the importance of centers experienced in gastric cancer surgery to perform an adequate lymphadenectomy with good long-term oncological outcome, without increasing postoperative complications or mortality, even in overweight or obese patients.

## 5. Conclusion

In conclusion, according to our data higher preoperative BMI is not related to postoperative outcome and long-term results in gastric cancer patients and therefore should not be considered a factor affecting surgical radicality, at least in specialized centers.

## Figures and Tables

**Table 1:** Clinicopathological features of normal (BMI < 25 kg/m^2^), overweight (BMI 25–30 kg/m^2^), and obese (BMI ≥ 30 kg/m^2^) patients.

	Total	Group A		Group B		Group C		*P* value
Patient (*n*)	378	218		121		39		
BMI (median)	24.03	22.05		27.00		31.62		<0.001
Age, y (median)	66	67.2		67		63		0.111 (Kruskal)
Sex (male, female)	223 : 155	116 : 102		84 : 37		23 : 16		0.015 (*χ* ^2^)
Diabetes (yes, no)	51 : 319	23 : 192		15 : 101		13 : 26		<0.001 (*χ* ^2^)
Missing	8							
pT								0.945 (*χ* ^2^)
1a	42	25	**11.5%**	13	**10.7%**	4	**10.3%**	
1b	30	17	**7.8%**	10	**8.3%**	3	**7.7%**	
2	53	25	**11.5%**	23	**19.0%**	5	**12.8%**	
3	72	44	**20.2%**	21	**17.4%**	7	**17.9%**	
4a	162	94	**43.1%**	50	**41.3%**	18	**46.2%**	
4b	18	12	**5.5%**	4	**3.3%**	2	**5.1%**	
Missing	1	1	**0.5%**					
pN								0.132 (*χ* ^2^)
0	136	81	**37.2%**	45	**37.2%**	10	**25.6%**	
1	39	16	**7.3%**	15	**12.4%**	8	**20.5%**	
2	51	35	**16.1%**	12	**9.9%**	4	**10.3%**	
3a	72	35	**16.1%**	28	**23.1%**	9	**23.1%**	
3b	78	49	**22.5%**	21	**17.4%**	8	**20.5%**	
Missing	2	2	**0.9%**					
Tumor site								0.514 (*χ* ^2^)
Upper third	53	28	**12.8%**	17	**14.0%**	8	**20.5%**	
Middle third	104	57	**26.1%**	39	**32.2%**	8	**20.5%**	
Lower third	193	113	**51.8%**	59	**48.8%**	21	**53.8%**	
Diffuse	16	10	**4.6%**	5	**4.1%**	1	**2.6%**	
Gastric stump	10	9	**4.1%**	0		1	**2.6%**	
Missing	2	1	**0.5%**	1	**0.8%**	0		
Lauren								0.318 (*χ* ^2^)
Diffuse	104	65	**29.8%**	24	**19.8%**	15	**38.5%**	
Intestinal	227	126	**57.8%**	82	**67.8%**	19	**48.7%**	
Mixed	35	20	**9.2%**	11	**9.1%**	4	**10.3%**	
Missing	12	7	**3.2%**	4	**3.3%**	1	**2.6%**	
Morbidity								0.803 (*χ* ^2^)
Yes	114	63	**28.9%**	38	**31.4%**	13	**33.3%**	
No	264	155	**71.1%**	83	**68.6%**	26	**66.7%**	
Major morbidity								0.696 (*χ* ^2^)
Yes	87	47	**21.6%**	31	**25.6%**	9	**23.1%**	
No	291	171	**78.4%**	90	**74.4%**	30	**76.9%**	
Postoperative death								0.263 (*χ* ^2^)
Yes	14	11	**5.0%**	2	**1.7%**	1	**2.6%**	
No	364	207	**95.0%**	119	**98.3%**	38	**97.4%**	
Hospital stay, days: median	11	12		12		11		0.356 (Kruskal)
Examined lymph nodes (median)	35	37		33		35		0.059 (Kruskal)
Metastatic lymph nodes (median)	4	4		3		5		0.586 (Kruskal)

**Table 2:** Summary of studies from Europe, Asia, and USA.

Year	Author	Country	BMI stratification	Cohort size	Outcome		
					Number of lymph nodes retrieved	Morbidity	Survival
2000	Inagawa et al. [23]	Japan	<20; 20–25; >25	293		No difference	No difference
2003	Gretschel et al. [28]	Germany	<25; 25–30; >30	199	No difference	No difference	
2004	Murphy et al. [37]	UK	<20; 20–25; 25–30; >30	50		No difference	
2008	Yamada et al. [31]	Japan	<25; >25	248	No difference	No difference	No difference
2009	Oh et al. [26]	Korea	<25; >25	410	Significant difference	Significant difference	No difference
2009	Tokunaga et al. [34]	Japan	<25; >25	7925			Significant difference
2009	Ojima et al. [36]	Japan	<25; >25	689		Significant difference	No difference
2011	Nobuoka et al. [35]	Japan	<25; >25	644		Significant difference	No difference (except for stage IV)
2010	Kulig et al. [9]	Poland	<25; >25	1992	Significant difference	No difference	Significant difference
2012	Lee et al. [38]	Korea	<25; >25	243	No difference	No difference	
2012	Oh et al. [39]	Korea	<25; >25	61	No difference	No difference	
2013	Bickenbach et al. [10]	USA	<25; >25	1853	Significant difference	Significant difference	No difference
2013	Pata et al. [27]	Italy	<25; 25–30; >30	161	Significant difference	Significant difference	
2013	Lin et al. [25]	Taiwan	<25, 25–30, >30	947	Significant difference	Significant difference	No difference
2014	Wong et al. [29]	USA	<18.5; 18.5–25; 25–30; >30	222	Significant difference	No difference	Significant difference
2014	Kim et al. [30]	Korea	<18.5; 18.5–23; 23–25; 25–30; >30	304	Significant difference	No difference	No difference
2014	**Present experience**	**Italy**	**<25; 25–30; >30**	**378**	**No difference**	**No difference**	**No difference**
